# Novel Barrier Enclosure for Both Aerosol and Droplet Protection Model

**DOI:** 10.5811/westjem.2020.6.47834

**Published:** 2020-06-29

**Authors:** Chad E. Branecki, Nicholas J. Jobeun, Tyler J. Ronnfeldt, Michael A. Ash, Thomas E. Schulte, Jason G. Langenfeld

**Affiliations:** * University of Nebraska College of Medicine, Department of Emergency Medicine, Omaha, Nebraska; † University of Nebraska College of Medicine, Department of Internal Medicine, Omaha, Nebraska; ‡ University of Nebraska College of Medicine, Department of Anesthesia, Omaha, Nebraska

## Abstract

Emergency physicians are on the front lines of treating patients with highly infectious respiratory diseases. Personal protective equipment is one defense against contamination from droplet and aerosol secretions. Intubation is a procedure that greatly can increase provider’s risk of exposure. Utilization of an intubation box has been discussed and recommended on social media platforms. There has been scant literature demonstrating the effectiveness of such devices. This study aimed to determine degree of droplet contamination to the intubator utilizing a novel barrier enclosure with a fluorescent simulated respiratory contagion. This model confirmed both added protection to the providers preforming intubation, and reduction of spread of the droplets when such a device is applied to patient care.

## INTRODUCTION

With the spread of severe acute respiratory syndrome coronavirus 2 (SARS-COV-2) worldwide, hospitals must provide equipment and strategies to protect frontline healthcare workers especially during procedures likely to generate aerosols and droplets. Hospitals that have implemented effective strategies to protect healthcare staff have shown low infection rates for healthcare workers.^1^ Studies of previous SARS viruses has shown that tracheal intubation is one of the highest risk procedures.^2^ COVID-19 patients frequently present in respiratory distress and often require emergent airway interventions, leading to high risk for exposure to droplet and airborne secretions to healthcare personnel performing pre-oxygenation, induction, and intubation.

The use of an “intubation box” or barrier may protect staff during intubation.^3^ A modified barrier was constructed from a 3D printed design and used by investigators. This intubation box was then modified by UNMC anesthesia staff. The design is easily manufactured from snowmobile windshield material at low a cost ($65–$100 US), allowing for easy assembly, and disinfection. Unique features include the compact size when folded, making it more portable and easier to store than other alternative boxes constructed from rigid materials. This intubation box is similar in function to the COVid aErosol pRotEction Dome (“COVERED”) developed at the University HospitalFrankfurt, Germany with differences in design.^4^

We performed a simulation exercise to characterize the difference in exposure to an individual using standard (PPE), both with and without the protection from the novel folding intubation box. In comparisons to the recent *New England Journal of Medicine* (NEJM) simulation study, our study aimed to compare both large droplets in a simulated cough set up and micro-droplets using an atomizer.

## METHODS

The intubation box is a rigid enclosure with two arm holes on either side for easy access by the intubator, as well as a small semicircle at the base to allow access for oxygen, suction and ventilator tubing. The box is clear, to allow easy visualization of the patient and equipment.

**Figure 1:**
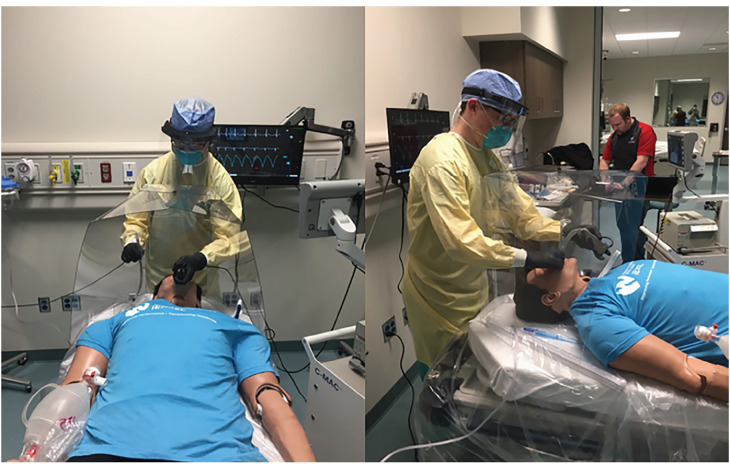
Demonstrations of intubation in proper PPE using intubation box. *PPE*, personal protective equipment.

We conducted three simulations to assess the effectiveness of the device, noting the difference in droplet and aerosol spread for each simulation. The investigators performed a control intubation with standard PPE including: gown, gloves, N95 mask, and face shield, but without the use of the intubation box. Second and third trials with similar PPE were performed using the intubation box used as a barrier to protect the user (Figure 1) in both simulated cough and atomized trials. The mannequin and intubator were decontaminated between all of the simulated trials.

**Figure 2:**
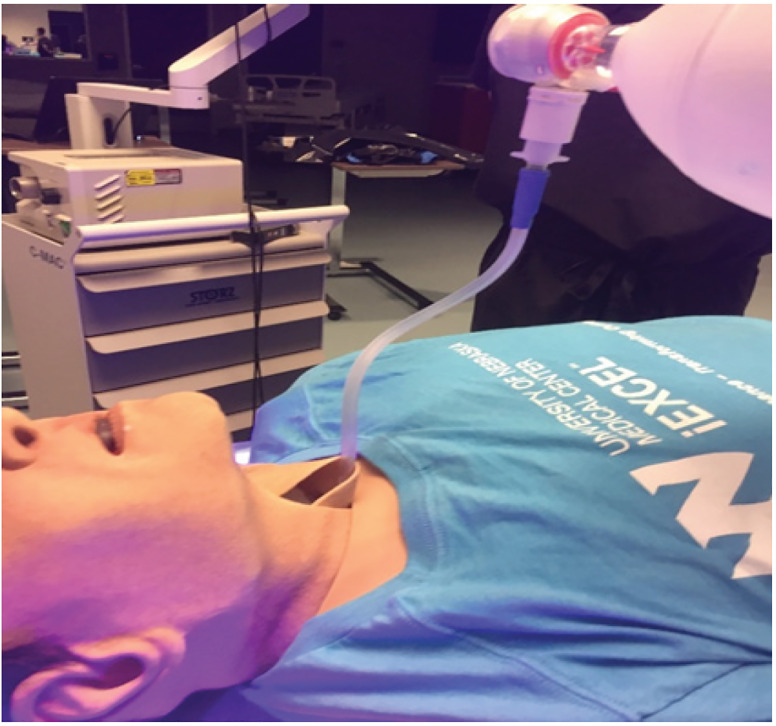
Large Droplet Cough Model using retrograde intubation technique allows for expelled secretions to come directly from mannequin’s mouth. BVM used to forcefully expel 5 mL of fluorescent solution. *BVM*, bag valve mask; *mL*, milliliter.

**Figure 3:**
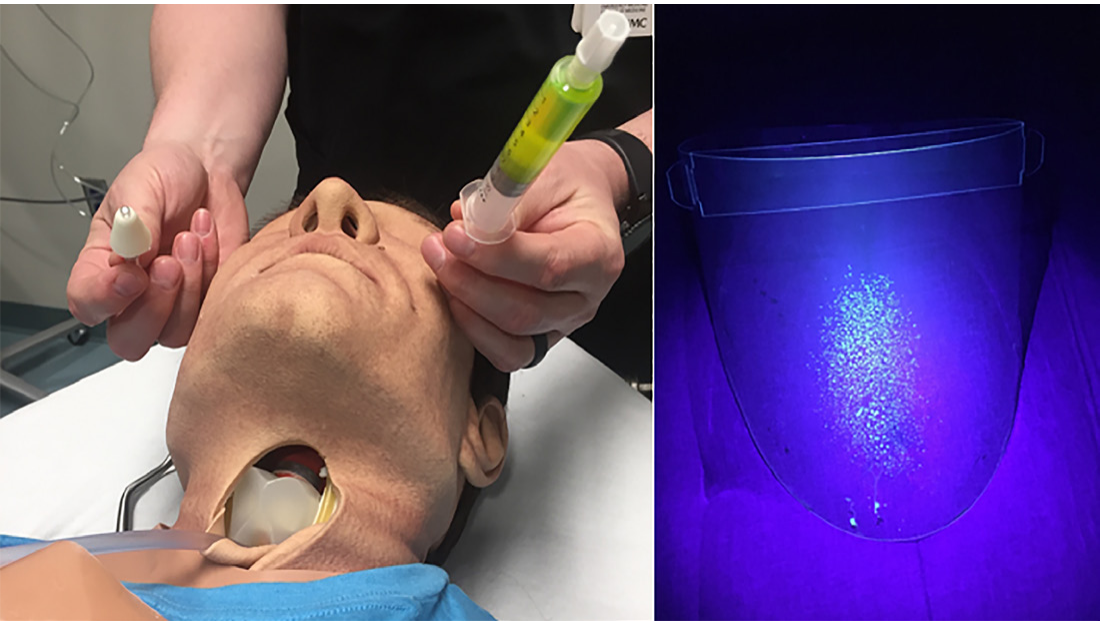
Micro Droplet Model using atomized 5 mL aliquot of fluorescein to replicate fine particle secretions. Intubator’s face shield post intubation without intubation box. *mL*, milliliter.

To simulate a cough, we instilled 5 mL of Glo Germ (Glo Germ Company, Moab UT), a fluorescent plastic particle, reconstituted in saline. The 5 mL filled syringe was attached to a bag valve mask (BVM) and a catheter was placed through the neck and into the oropharynx of the high-fidelity mannequin in a retrograde intubation fashion (Figure 2). Using a single hand, the BVM was used to simulate a forceful cough expelling a 5mL volume. Additionally, to generate aerosolization and micro-droplets, a second device was used by attaching the 10 mL filled syringe to an atomizer. To replicate micro droplet dispersal, another 5 mL of fluorescein dye was then atomized (Figure 3). Of note, plastic particle suspension was not used as it was too viscous to successfully atomize. The use of both devices provided replication of both fine and coarse droplets and aerosol spread. After each simulation, an LED black light was used to visualize the spread of the droplets by visualizing the fluorescent dye.

## RESULTS

**Figure 4:**
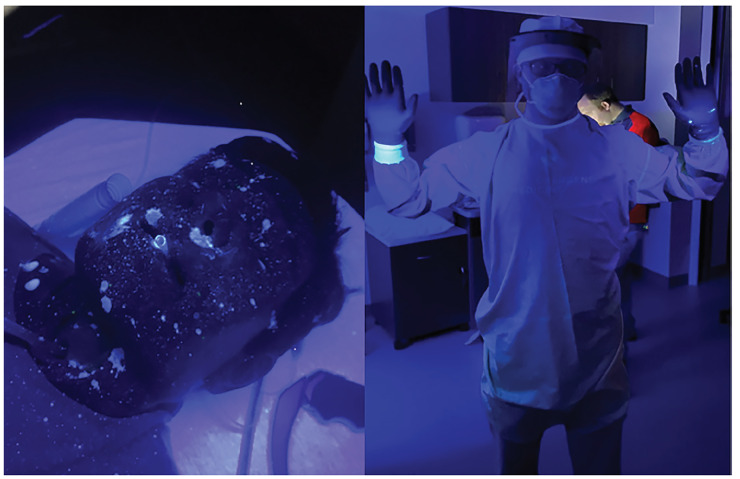
Despite heavy contamination of mannequin with large droplet cough using the intubation box; note only limited exposure to intubator’s hand that was inside of intubation box. No contamination on intubator’s face or torso.

**Figure 5:**
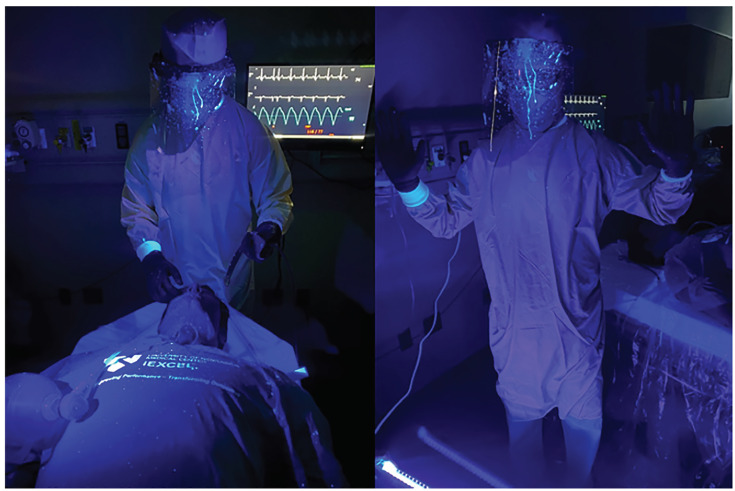
Extensive contamination on intubator’s head, and face shield when intubation box was not utilized during a simulated large droplet cough.

**Figure 6:**
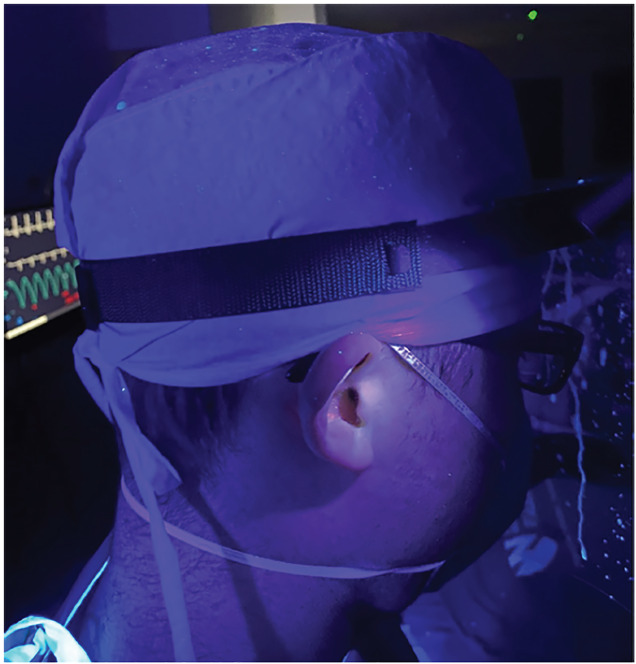
Note additional contamination on intubator’s surgical cap ties, and exposed ear during cough simulation when intubation box was not used. Increasing risk of exposure during doffing procedures.

**Figure 7:**
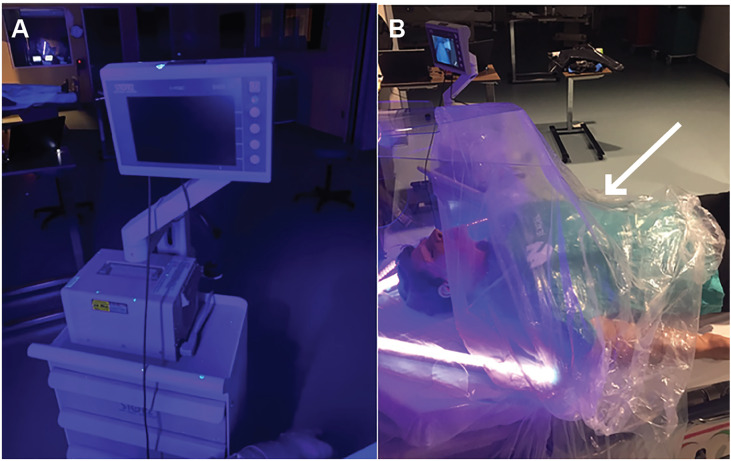
Panel A: Not just the laryngoscope blade, but the video laryngoscope screen and cart were also contaminated when intubation box was not employed. Panel B: Modifications to novel intubation were made to reduce the spread of droplets and contamination of room and equipment by adding clear surgical drape to the caudal end of the intubation box (arrow).

Comparing the area of simulated contamination between our control and experimental models demonstrated marked reduction in spread of fluorescein droplets when using the intubation box. With use of the intubation box, we effectively reduced the contamination of the proceduralist (Figure 4), with exposure limited to the proceduralist’s hands and PPE exposed inside the intubation box only and no identified contamination of PPE outside of the enclosure. Spread of fluorescent dye inferiorly onto the patient’s chest and lower extremities of greater than 4 feet did occur. In contrast, performing intubation without the intubation box resulted in significant contamination of both the intubator and the room. (Figure 5). The intubator had two areas of exposure that were not covered by PPE, one on the ear and one on the neck (Figure 6). The amount of droplet spread around the room was more than 6 feet without the box and with multidirectional distribution. The video laryngoscope and other equipment outside of the enclosure also showed contamination when the intubation box was not utilized (Figure 7A). Due to the small droplet size from the atomizer, we noticed less spread than the forceful large droplet cough.

**Population Health Research Capsule**

What do we already know about this issue?

*Health care workers are put at risk when preforming droplet and aerosol generating procedures. The amount of exposure can be reduced by donning proper personal protective equipment*.

What was the research question?

Would a novel barrier device add additional protection to health care workers from droplets and micro-droplet contamination?

What was the major finding of the study?

*The intubation box was effective in reducing the amount of direct exposure to simulated respiratory secretions*.

How does this improve population health?

*Not only would this protect the health care workforce individually, but could have the potential to reduce community spread of asymptomatic highly infectious respiratory diseases*.

## DISCUSSION

This simulation demonstrated several important findings pertaining to the protection of healthcare staff during intubation. The intubation box was effective in reducing the amount of direct exposure to simulated respiratory secretions that reached the intubator during a simulation of droplet and aerosol generation during intubation. In addition, the increased exposure to secretions on the proceduralist’s PPE without the intubation box leaves them more vulnerable to being exposed after the procedure by imperfect doffing. The box also decreased the amount of simulated secretions that was spread around the room. The majority of the spread of fluorescent droplets were caudal in location. The box is left open for the patient’s torso, intravenous support and monitor lines. Our study also showed that proper PPE use is effective in helping to protect the proceduralist from direct exposure from a cough during intubation without a barrier. Overall, the intubation box provided additional protection for healthcare providers during procedures that are high risk to generate aerosols and potentially spread infectious particles, such as intubation.

## LIMITATIONS

Some limitations to our simulation were effectively simulating the aerosolization of all secretions from a cough. It was difficult to model this accurately in the simulation lab, and therefore difficult to characterize the box’s effect on the generation of the smallest microscopic particles. With the accuracy of the droplet and aerosol simulation difficult to achieve, we are unable to quantify the results. Computerized modeling could be used for quantification and confirmation.

In addition, the box does make the intubation procedure somewhat more technically difficult, as the hand holes restrict freedom of movement of the intubator’s arms during the procedure. Current successful use in our hospital’s operating rooms shows that the box allows for safe intubation, but considerations should be taken before use including proper training in the procedure and achieving familiarity with the box prior to implementation. The effect experience with the intubation box has on the spread of contaminating droplets is an opportunity for further assessment.

Our model effectively demonstrated the spread of particles with forcible turbulent airflow, as seen with a cough (see supporting slow motion video in the digital format). However, a productive cough was difficult to replicate accurately with attention to velocity, viscosity, and volume of fluid. The utility of this model is more in identifying protection from respiratory secretion exposure. Modifications of the box with a tapered end or even placing a surgical drape at the caudal end of the box may provide additional protection from droplet spread (Figure 7B).

## CONCLUSION

One future application for this box could be as a tool to help protect providers who are administering nasopharyngeal swabs to test for SARS-COV-2. The hand ports provide easy access to the patient, while the barrier would help protect the healthcare provider from direct droplet exposure. This is especially important in swab collection procedure requires the patient to remove their mask, and the noxious stimulation of the swab makes the patient more prone to cough, sneeze or gag.

Overall, the use of the novel folding intubation box may prove useful in decreasing the spread of droplet contamination while performing intubation on patients with suspected highly infectious respiratory diseases. Further investigations into the mitigation of airborne particles, as well as other improvements, should be considered by physicians around the world to create innovative solutions to the problem of protecting healthcare workers worldwide during the SARS-COV-2 pandemic.

## Supplementary Information
